# TopBP1 and Claspin contribute to the radioresistance of lung cancer brain metastases

**DOI:** 10.1186/1476-4598-13-211

**Published:** 2014-09-12

**Authors:** Seung Ho Choi, Heekyoung Yang, Seung Ho Lee, Joo-Hyun Ki, Do-Hyun Nam, Hae Yong Yoo

**Affiliations:** Department of Health Sciences and Technology, Samsung Advanced Institute for Health Sciences and Technology, Sungkyunkwan University, 50 Ilwon-Dong, Gangnam-Gu, Seoul, 135-710 South Korea; Samsung Biomedical Research Institute, Research Institute for Future Medicine, Samsung Medical Center, Seoul, Korea; Cancer Stem Cell Research Center, Samsung Medical Center, Seoul, Korea; Department of Neurosurgery, Samsung Medical Center, Sungkyunkwan University School of Medicine, 50 Ilwon-Dong, Gangnam-Gu, Seoul, 135-710 South Korea

**Keywords:** Radiosensitivity, Lung cancer, Brain metastasis, TopBP1, Claspin

## Abstract

**Background:**

Radiation therapy is one of the most effective therapeutic tools for brain metastasis. However, it is inevitable that some cancer cells become resistant to radiation. This study is focused on the identification of genes associated with radioresistance in metastatic brain tumor from lung cancer and the functional examination of the selected genes with regards to altered sensitivity of cancer cells to radiation.

**Methods:**

After establishing radioresistant cells from the xenograft model, we explored the significant transcriptional changes by performing DNA microarray profiling. Functional analyses *in vitro* and *in vivo* performed to validate the gene responsible for radioresistance.

**Results:**

Transcriptional changes induced by radiation therapy are much more extensive in H460 cells than in PC14PE6 cells. The expression levels of TopBP1 and Claspin were increased in the cancer cells that survived radiation therapy. Depletion of TopBP1 or Claspin using shRNA showed an enhancement of sensitivity to radiation in radioresistant lung cancer cells (PC14PE6). Moreover, increased levels of TopBP1 or Claspin endowed cells a higher resistance to radiation. In xenograft models, the knock-down of TopBP1 or Claspin significantly prolonged the median survival time post radiation therapy.

**Conclusions:**

We analyzed the gene expression profiles of the radiosensitive cells and the radioresistant cells to define a set of genes that may be involved in endowing lung cancer cells radioresistance post brain metastasis. Functional analyses indicated that the expression TopBP1 and Claspin positively affects the survival of cancer cells and thus negatively the xenograft metastasis model animals in response to radiation. These results show that TopBP1 and Claspin can be potential targets for the enhanced efficacy of radiotherapy.

**Electronic supplementary material:**

The online version of this article (doi:10.1186/1476-4598-13-211) contains supplementary material, which is available to authorized users.

## Background

Brain metastases occur in 20 to 40% of all patients with cancer [1]. The problem is particularly challenging for lung cancer patients as they often present with multiple brain metastases. Even though multiple chemotherapeutic agents have been studied for treatment of metastatic brain tumors (MBTs), their clinical availability and effectiveness are still limited. In these circumstances, whole-brain radiation therapy has been used in the management of brain metastases [1]. In fact, radiation therapy or stereotactic radiosurgery often turn out to be the only available tools in the cases of multiple MBTs. However, the frequent resistance to radiation resulting in recurrence leads to treatment failure in a substantial fraction of MBT patients.

Recently, various checkpoint mechanisms have received much attention in association with genetic damages from UV light or ionizing radiation (IR). DNA damage checkpoints are important parts of signal-transduction pathways that delay or arrest cell cycle progression in response to the damage. Originally, checkpoints aid maintaining genomic integrity and cell survival. However, these mechanisms can be used by cancer cells to evade apoptosis from DNA injury, resulting in acquiring resistance to irradiation [2, 3]. There are several reports suggesting that the checkpoint can be exploited to enhance radiosensitivity [4, 5]. As a mediator, topoisomerase IIβ binding protein 1 (TopBP1) is an essential co-activator of ATR [6]. In addition, this protein has a direct and essential role in the pathway that connects ATM to ATR [7]. Claspin was identified as a Chk1-interacting protein and an important regulator of Chk1 activation [8, 9].

Cells with functionally defective components of DNA damage checkpoint pathways show cell cycle checkpoint defects, and an increased sensitivity to IR and other DNA damage agents [2, 10]. This latter observation highlights components of these DNA damage checkpoint pathways as potential therapeutic targets for enhancing the sensitivity of tumor cells to the radiotherapeutic agents [11]. Tumor cell specific checkpoint mechanisms for DNA damage in response to IR can thus provide clues to problems of radioresistance.

In the present study, we sought to genes associated with radioresistance in metastatic brain tumor and to investigate how they can be targeted for enhanced susceptibility of carcinoma cells to radiation. We analyzed the gene expression profiles of lung cancer cells with and without radiation treatment using xenograft models of lung cancer brain metastasis. We found that the expression levels of TopBP1 and Claspin were significantly increased in the surviving cancer cells after radiation therapy compared to untreated cells. Consistently, depletion of TopBP1 or Claspin in lung cancer cells showed an enhancement of sensitivity to radiation. In xenograft models, the knock-down of TopBP1 or Claspin significantly prolonged the median survival time in response to radiotherapy. Therefore TopBP1 and Claspin are potential therapeutic targets for overcoming the limitation of radiotherapy for MBT.

## Results and discussion

### Induction of radioresistance using lung cancer brain metastases xenograft

**Figure 1:**
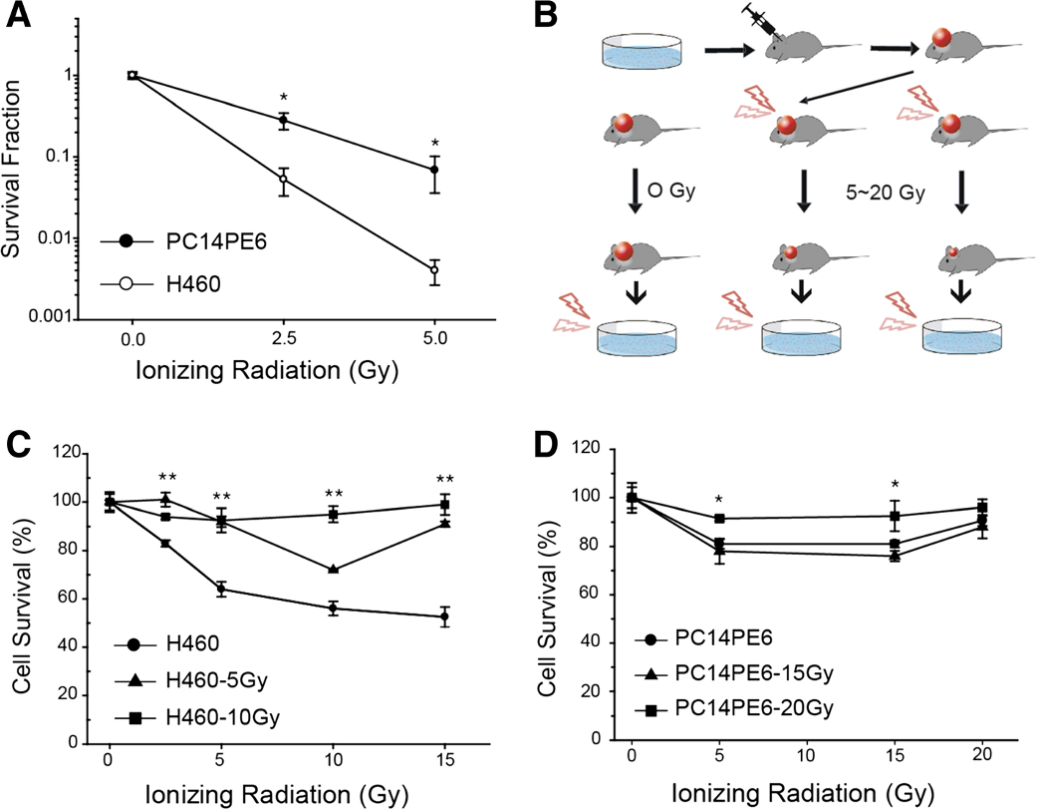
**Induction of radioresistant cells** ***in vivo*** **. (A)** Clonogenic survival assays were performed with ionizing radiation (IR) treatment as indicated. Two lung cancer cell lines (PC14PE6 and H460) were exposed to IR and the survival was measured. **(B)** Cartoon shows the procedure for the generation of radioresistant lung cancer cells in lung cancer brain metastasis xenograft model. Each lung cancer cells (PC14PE6 and H460) were xenografted into a mouse brain to produce orthotopic metastasis model animals which were irradiated at various doses (H460, 5 and 10 Gy; PC14PE6, 15 and 20 Gy). **(C-D)** The H460 **(C)** and PC14PE6 **(D)** cells from the mouse brain were cultured *in vitro*, and exposed to various doses of radiation as indicated. After exposure to IR, cell survival was evaluated by cell proliferation assay (CCK-8 assay). Values are from at least three independent experiments and error bars represent the standard deviation. *P-value < 0.05; **P-value < 0.005.

We assessed radiosensitivity of lung cancer cell lines (PC14PE6 and H460) with various doses of radiation. The clonogenic survival assays revealed that both cell lines showed clear radiation sensitivity in a dose-dependent manner, and the survival fraction of PC14PE6 showed 0.4 at 2.5 Gy whereas the survival fraction of H460 cells was more severely reduced down to 0.04 at 2.5 Gy (Figure 1A). These reduced radiation sensitivity of PC14PE6 indicated that PC14PE6 cells were relatively more resistant to radiation than H460 cells. We made lung cancer brain metastases xenograft models using PC14PE6 and H460 cells and tested whether tumor cells in xenograft models acquired radioresistance after radiotherapy. As shown in Figure 1B, xenografted mice models were exposed to radiation once, and the tumor cell mass was excised and cultured *in vitro* again. To validate whether radiosensitivity of tumor cells were changed, the tumor cells were irradiated with a range of radiation doses (5–20 Gy). Survival representing resistance to radiation was examined by cell proliferation assay and shown by percent of viability to non-irradiated cells (Figure 1C & D). Initially H460 cells have been less resistant to radiation, but cells from irradiated tumor cells (H460-5Gy and H460-10Gy) acquired radioresistance of over 90% of the survival rate (Figure 1C). H460-10Gy cells were even more radioresistant than H460 and H460-5Gy. These results show that radiosensitive H460 cells acquired radioresistance in a dose dependent manner. However, PC14PE6, originally more radioresistant than H460 did not show additional increase of resistance regardless of the radiation dose (Figure 1D).

### Alteration in gene expression profiles

**Figure 2:**
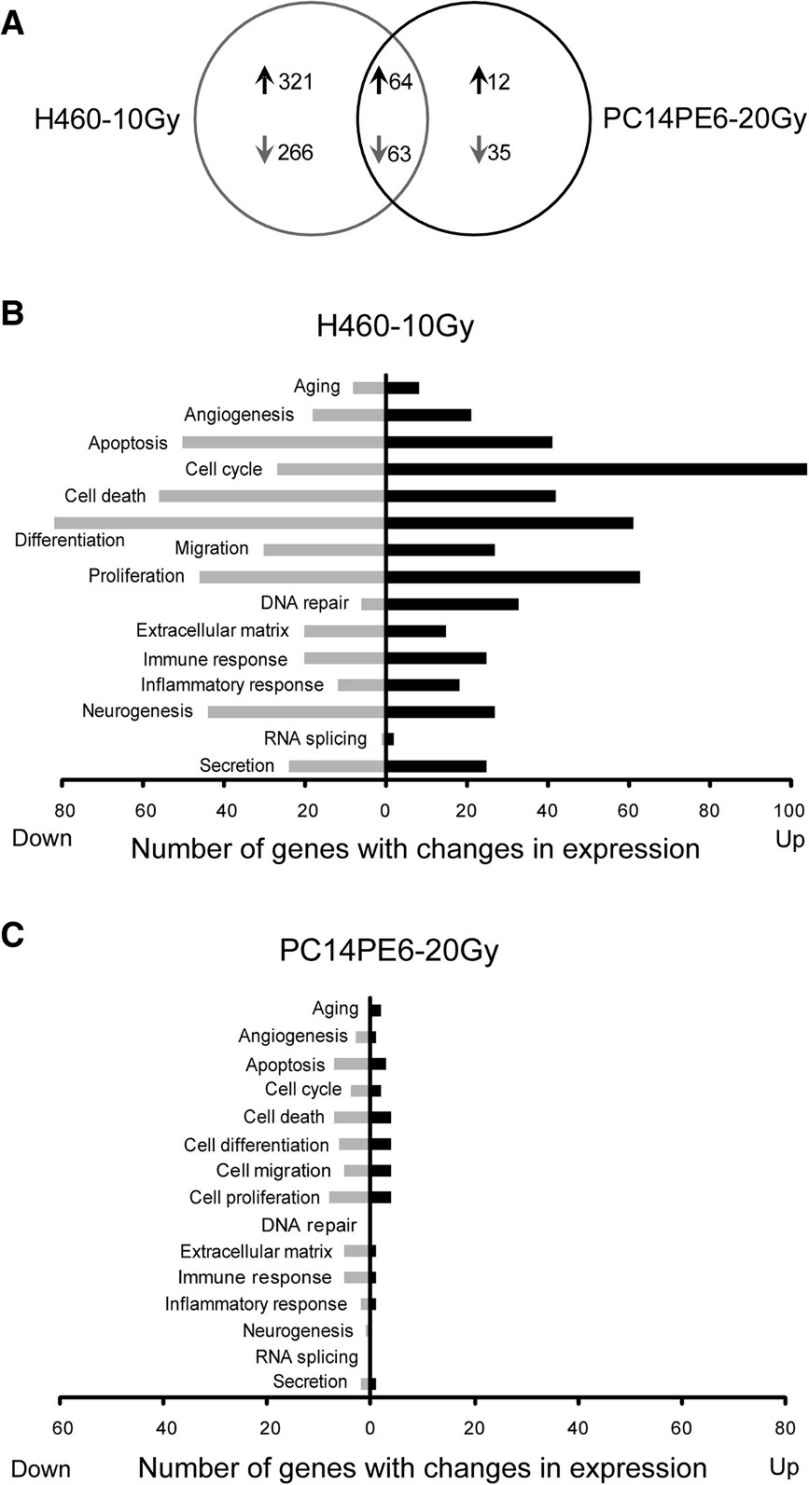
**Expression microarray analysis of two induced radioresistant cell lines. (A)** Venn diagram shows the number of genes with expression change by radiotherapy in PC14PE6 or H460 cells from xenografted models. Genes were selected using a filter criterion of at least 2-fold change compare to no radiotherapy control with *p* < 0.05. **(B-C)** Genes with altered expression in radioresistant H460-10Gy and PC14PE6-20Gy are categorized into 15 functional groups based on the gene ontology. The gene numbers are displayed in graphs.

After establishing radioresistant cells from the xenograft model, we explored the significant transcriptional changes by performing DNA microarray profiling. Using a filter criterion of at least 2-fold change with *p* < 0.05, the numbers of genes with changes in expression in H460-10Gy and PC14PE6-20Gy cells were determined: 385 genes increased and 329 genes decreased in H460 while 76 genes increased and 98 genes decreased in PC14PE6. Between the two cell lines, 64 of the increased genes and 63 of the decreased genes overlapped. These results are presented in a Venn diagram (Figure 2A). In particular, the number of affected genes in H460-10Gy cells was 4.1 fold higher than that in PC14PE6-20Gy. These results showed that transcriptional changes induced by radiation therapy are more extensive in radiosensitive cells (H460) than in resistant cells (PC14PE6). Functional annotation of the genes was carried out using gene ontology-based biological property analysis. The genes were categorized into 15 functional groups (Figure 2B & C). Most of the changed genes in the two cell lines were associated with cell differentiation, proliferation and cell cycle. The genes with significant changes in expression are listed in Additional file 1: Table S1-S4.

### Enhanced radiosensitivity from depletion of TopBP1 and Claspin

Genes involved in cellular response to DSBs are the major targets for cancer cell death [12]. Specifically, functional defects in DNA damage checkpoint pathways show an increased sensitivity to radiation [2, 10, 13], which led us to focus on the DNA Damage response (DDR) signaling pathway for subsequent analyses. The genes with expression change in DNA damage checkpoint pathway are listed in Additional file 1: Table S5. Notably, genes from H460-10Gy outnumber those from PC14E6-20Gy. We chose TopBP1 and Claspin genes as our targets for functional analysis as they play important roles for activating Chk1 in DDR pathway [6, 8, 14]. In particular, since Chk1 inhibition using inhibitors showed an enhancement of radiation sensitivity [13, 15], it is worth analyzing the role of genes upstream to Chk1 with regards to radiosensitivity. Chk1 is a well-established ATR target involved in DDR and normal cell cycle progression and thus represents a potential pharmacological target in the development of anti-cancer therapeutic agents [16, 17].

**Figure 3:**
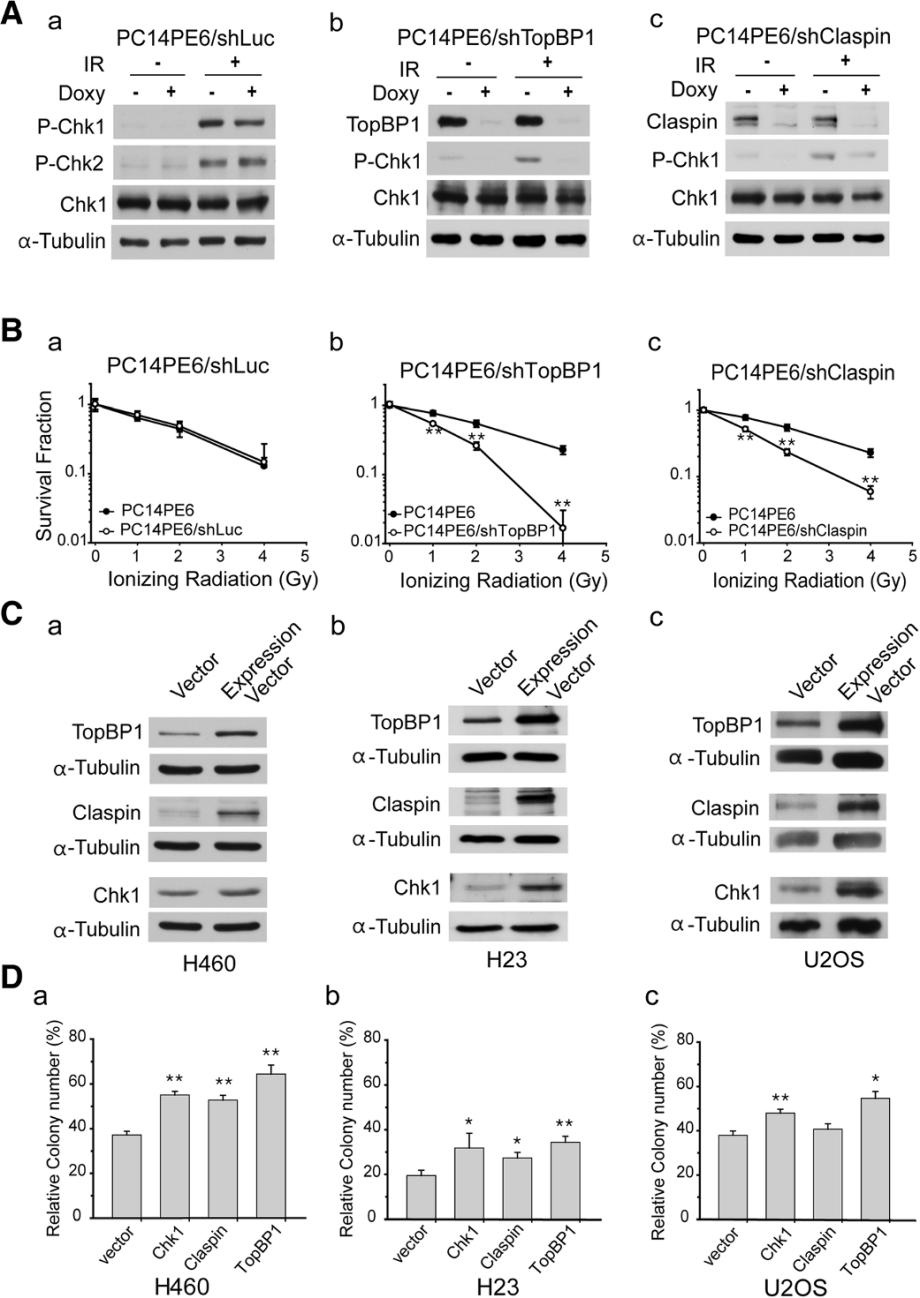
**Depletion of TopBP1 or Claspin enhances radiosensitivity of PC14PE6 cells** ***in vitro*** **. (A)** TopBP1, Claspin, and luciferase knock-down cells are regulated by doxycycline treatment through Tet On/Off system. The cells expressing luciferase shRNA were used as a control. To knockdown the target genes, the radio-resistant PC14PE6/shRNA cells were treated with doxycycline (1 μg/ml) for 72 hours and exposed to 10 Gy of IR. The protein levels were evaluated by Western blotting. The phosphorylation of Chk1 S317 and Chk2 Thr68 were induced by exposure of radiation. TopBP1 and Claspin protein levels were significantly down-regulated with the treatment of doxycycline. **(B)** Cell survival was measured by clonogenic survival assay in TopBP1 or Claspin down-regulated stable PC14PE6 cells with IR treatment as indicated. **(C)** For overexpression of TopBP1, Claspin, or Chk1, overexpression plasmids were transfected into H460 (a), H23 (b), and U2OS (c) cells. The expression of each protein was shown by Western blot. α-tubulin was used as the loading control. **(D)** Overexpression of Chk1, TopBP1 or Claspin enhances radioresistance *in vitro*. Expression vector of each gene was transfected into three kinds of cell lines. The transfected (a) H460 cells, (b) H23 cells, and (c) U2OS cells were irradiated at 2 Gy. Radiosensitivity was evaluated by clonogenic assay. Each experiment in triplicates was repeated three times. Error bars represent the standard deviation. *P-value < 0.05; **P-value < 0.005.

To confirm and validate the findings obtained from our microarray analysis, we used the radioresistant PC14PE6 cells and established stable lines in which TopBP1 and Claspin were regulated by shRNA via doxycycline treatment. It was confirmed that the mRNA levels of the two genes decreased by doxycycline treatment compared to untreated PC14PE6 cells (Additional file 1: Figure S1A & B). Induction of shRNA virtually completely abolished the expression of TopBP1 and Claspin (Figure 3A). It has been shown that TopBP1 and Claspin play an essential role in Chk1 activation/phosphorylation in response to DNA damage [6, 14]. We next examined the phosphorylation of Chk1 in response to irradiation after knockdown of TopBP1 and Claspin. As shown in Figure 3A, depletion of TopBP1 or Claspin abolished Chk1 phosphorylation as previously reported [6, 7, 14]. Immunocytochemistry of γ-H2AX and TopBP1 demonstrated that γ-H2AX foci were significantly decreased and the signals for TopBP1 were scantly detected after the depletion of TopBP1 (Additional file 1: Figure S1C).

We performed clonogenic survival assay to measure the change in sensitivity to radiation in these stable cell lines. The PC14PE6/shLuc cells showed a similar sensitivity to irradiation in comparison to original PC14PE6 cells (Figure 3Ba). TopBP1 and Claspin knockdown cells showed a higher sensitivity than control cells. The decrease in survival was particularly dramatic at higher dose of IR (e.g. 4 Gy) than at lower dose of IR (< 2 Gy) for both PC14PE6/shTopBP1 and PC14PE6/shClaspin cells (Figure 3Bb & c). Our results are consistent with a previous report describing an enhanced susceptibility to radiation in cells with down-regulated Chk1 [13].

### Enhanced radioresistance in cells from overexpression of TopBP1 or Claspin

The expression levels of TopBP1 and Claspin increased in induced radioresistant H460-10Gy cells. To clarify and further evaluate their roles with radioresistance, TopBP1, Claspin, and Chk1 were overexpressed in three independent cell lines, H460, H23, and U2OS which are all highly radiosensitive compared to PC14PE6 (Figure 3C & Additional file 1: Figure S2). The overexpression of each gene was examined by immunoblot (Figure 3C). In clonogenic assay, GFP expression vector was used as a control. In H460 and H23 cells, overexpressing TopBP1 showed higher survival (over 50%) than control cells after 2 Gy IR exposure (Figure 3D). In U2OS, the number of surviving colonies increased by about 20% compared to the control. These results indicate that overexpression of TopBP1, Claspin, and Chk1 induces radioresistance, consistent with the microarray data.

### Extended survival of brain metastasized mice by TopBP1 or Claspin knockdown

**Figure 4:**
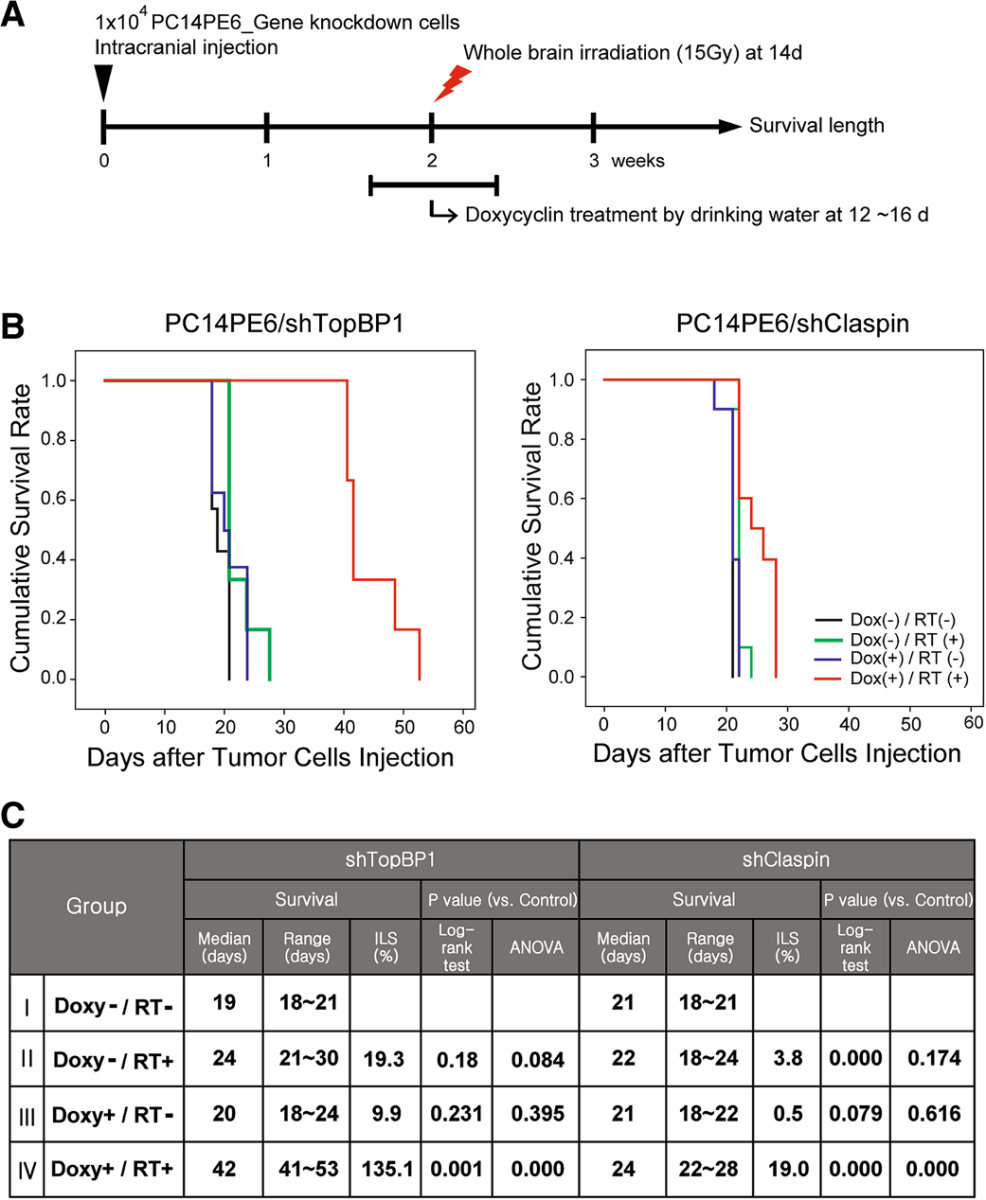
**Knockdown of TopBP1 or Claspin extended survival of brain metastasized mice. (A)** Schedule of treatment. Whole brain irradiation (15 Gy) was performed after 2 weeks. TopBP1 or Claspin expression was inhibited for 5 days with doxycycline in drinking water at a dose of 1 mg/kg from day 12. **(B)** Kaplan-Meier plot comparing survival of PC14PE6/shTopBP1 (n = 7 for control, n = 6 for sham-irradiated and combination group, and n = 8 for shTopBP1 group) (a) and PC14PE6/shClaspin (n = 10 for each group) (b) orthotopic brain metastases models with indicated treatments. **(C)** The table presents the survival time of the mice that were injected with TopBP1 or Claspin down-regulated tumor cells under indicated conditions.

Based on results from *in vitro* functional assays, we hypothesized that TopBP1 or Claspin would be potential target genes in lung cancer brain metastasis models (Figure 4). We thus sought to estimate the effects of TopBP1 and Claspin knockdown based on the survival of lung cancer brain metastasis xenograft model animals in response to irradiation. We produced orthotopic xenograft animals with injection of PC14PE6/shTopBP1 or PC14PE6/shClaspin cells into the brain of athymic nude mice. Tumor-bearing mice were treated with doxycycline and/or radiation therapy as illustrated (Figure 4A). As shown in Figure 4B, without irradiation, there was no difference in survival rates between TopBP1-inhibited (i.e. doxycycline-treated) group and TopBP1-expressing group. The median survival time was 19 days. In contrast, when irradiated, xenografted mice with inhibited expression of TopBP1 showed a higher survival rate than the control mice with the mean life span of 42 days post injection (Figure 4C). In case of Claspin knockdown mouse, the survival also extended with irradiation and doxycycline treatment although the effect was not as dramatic as in the case of TopBP1 knockdown. Lung cancer brain metastasis models in this study do not represent patients completely. Radiotherapy did not mimic patient dosing and fractionation schedules for limitation of animal models. In sum, xenotransplant analysis further confirmed the proposed role of TopBP1 or Claspin in DDR response and acquisition of radioresistance.

## Conclusions

In this study, we comparatively analyzed the gene expression profiles of the radiosensitive cells and the radioresistant cells to define a set of genes that may be involved in endowing lung cancer cells radioresistance post brain metastasis. Functional analyses *in vitro* and *in vivo* indicated that the expression TopBP1 and Claspin positively affects the survival of cancer cells and thus negatively the xenograft metastasis model animals. This is consistent with results from our previous study which demonstrated elevated expression levels of DNA damage checkpoint genes in radioresistant lung cancer cell lines compared to the levels in radiosensitive cell lines [18]. The expression pattern of DNA damage checkpoint genes thus could be useful for diagnosis and prognosis of brain metastasis and be promising targets for overcoming the radio-resistance in brain metastasis.

## Methods

### Cell culture and cell lines

Human non-small cell lung cancer (NSCLC) cell lines H23, H460, and human osteosarcoma cell (U2OS) (American Type Culture Collection) were maintained in RPMI 1640 medium containing 10% fetal bovine serum, 100 U/ml penicillin, and 100 μg/ml streptomycin, and PC14PE6, kindly provided by MD Anderson Cancer Center was maintained in Eagle’s minimal essential medium (MEM) containing 10% fetal bovine serum, 100 U/ml penicillin, and 100 μg/ml streptomycin at 37 °C and 5% CO_2_. Cell lines were regularly tested for mycoplasma infection using the MycoAlert mycoplasma detection kit (Lonza). Isolated PC14PE6 and H460 cells from xenograft mouse brains were immediately used to assay for the cell viability after dissociation procedure.

### Transfection and stable cell lines

For overexpression of TopBP1, Claspin, and Chk1 in H23, H460 or U2OS cell lines, each cell lines were transfected with expression vectors by electroporation using Cell Line Nucleofector® Kit V with the Amaxa Nucleofector I system (Lonza). Stable cell lines of tetracycline-regulated expression of a short hairpin RNA were generated as previously described [13]. For shRNA induction, cells were seeded to achieve 30%-50% confluency on the day of induction and doxycycline was added into the culture medium at 1 μg/ml.

### Western blotting

Cells were lysed and prepared for Western blotting analysis as previously described [19]. Anti-Chk1 and anti-α-tubulin antibodies were purchased from Santa Cruz Biotechnology. Anti-Claspin, anti-Chk1 phospho-Ser317, anti-Chk2 phospho-Thr68 and anti-γ-H2AX antibodies were obtained from Cell Signaling Technology. Anti-TopBP1 was obtained from Abcam. The secondary antibodies Cy3-conjugated anti-mouse IgG and FITC-conjugated anti-rabbit IgG for immunofluorescence were purchased from Jackson Immunoresearch.

### RNA interference

siRNAs were obtained from Genolution Pharma, Korea. The sequences of the siRNAs targeting TopBP1 were: #1, CAGAAUUGUUGGUCCUCAAdTdT and #2, CGAUAGAGGAGACUCAUGAdTdT. The sequences of Claspin siRNA 1 and siRNA 2 were GGAAAGAAAGGCAGCCAGAdTdT and CCUUGCUUAGAGCUGAGUCdTdT, respectively. The control luciferase siRNA sequence was GTTACGCTGAGTACTTCGAAA. After the test of mRNA knockdown efficacy, plasmids expressing shRNA were constructed by shRNA cloning service from Genolution.

### Cell proliferation assay and clonogenic survival assay

Cell proliferation was analyzed with Cell Counting Kit 8 (Dojindo Molecular Technology) and clonogenic survival assay was performed as previously described [13]. Cell proliferation was expressed as a percentage of the absorbance obtained in irradiated cells relative to that in untreated control cells. Each experiment in triplicates was repeated three times. Error bars represent the standard deviation.

### Orthotopic lung cancer brain metastases xenograft studies

All experiments were conducted in accordance with the Institute Laboratory Animal Research Guide for the Care and Use of Laboratory Animals and within the protocols approved by the appropriate Institutional Review Boards at the Samsung Medical Center (Seoul, Korea). Female athymic nude mice, 7 weeks of age were used for this study. Production of orthotopic lung cancer brain metastases models were performed as previously described [13]. Mock-irradiated or irradiated mice were anesthetized with 100 mg/kg ketamine and 10 mg/kg xylazine and restrained in custom-designed mice jig. Whole brain irradiation (15 Gy) was performed after 2 weeks. To induce knockdown of TopBP1 and Claspin, doxycycline, which was formulated in 5% sucrose, was administered for 5 days in drinking water from 12 days after tumor cells implantation at a dose of 1 mg/ml.

### RNA expression array and data analysis

RNA expression array was performed as previously described [18]. RNA was transcribed into cDNA and hybridized onto Affymetrix GeneChip HG-U133Plus2.0 according to the Affymetrix GeneChip Expression Analysis Technical Manual. The gene expression CEL files were normalized using the Robust Multichip averaging procedure. The dataset was normalized using Central Tendency Median Normalization. Expression array data were deposited at http://tbi.skku.edu/microarray/smc/yoo-20140106.

### Statistical analysis

The survival analysis of xenograft model was performed using the Kaplan-Meier and log rank tests (SPSS statistical software, version 18.0). Increasing life span (ILS, %) compared to median survival of negative control was calculated, and statistical comparison was analyzed with one-way analysis of variance (ANOVA) followed by the lease significant difference (LSD) test. In vitro experimental data were obtained from experiments repeated three times with triplicates. Mean values were calculated and significance was determined using the Student’s two tailed test. *P* values < 0.05 were considered statistically significant.

## Electronic supplementary material

Additional file 1: **Supplementary Methods, Table S1-S5, and Figure S1-S2.** (DOCX 2 MB)

## Abbreviations

MBT: Metastatic brain tumor
IR: Ionizing radiation
TopBP1: Topoisomerase IIβ binding protein 1
DDR: DNA damage response.
