# Supplementary material for: TopBP1 and Claspin contribute to the radioresistance of lung cancer brain metastases
Source: Mol Cancer. 2014 Sep 12;13:211. doi: 10.1186/1476-4598-13-211 (PMC4168047; doi:10.1186/1476-4598-13-211)

**Additional file 1**

**TopBP1 and Claspin contribute to the radioresistance of lung cancer brain metastases**

**Additional file 1: Methods**

**Detection of mRNA for down-regulated genes by RT-PCR**

Total RNA was extracted, and Q-PCR was performed using power SYBR Green Cells to CT Kit (Invitrogen) according to the manufacturer’s instructions. For quantification the 2-ΔΔC_T_ method was used to determine the relative expression level of each gene The primers used are : foward primer 5’-tccgcaccctagagaaaaga-3’ and reverse primer 5’-aaggtaggagcgatgaagca-3’ for TopBP1; forward 5’-ATGCTTCCCAGATGGACTTG-3’ and reverse 5’- AGCCACTGCTCTCGTTCAAT-3’ for Claspin; forward 5’-GAAGGTGAAGGTCGGAGTC-3’ and reverse 5’-GAAGATGGTGATGGGATTTC-3’ for GAPDH.

**Immunofluorescence staining**

Cells grown on chamberslide (Nunc) were irradiated at 10 Gy. After 2 hours, cells were fixed in 4% paraformaldehyde for 10 min and then permeabilized in 0.25% Triton X-100 containing solution for 10 min. For immunostaining, cells were incubated with primary antibodies diluted in 5% goat serum at 37°C for 30 min. Cells were washed three times with PBS and then incubated with either fluorescein (FITC)-conjugated or Cy3-conjugated secondary antibodies at 37°C for 1 hour. Nuclei were counterstained with DAPI. Confocal images were acquired using a Zeiss LSM 700 microscope.


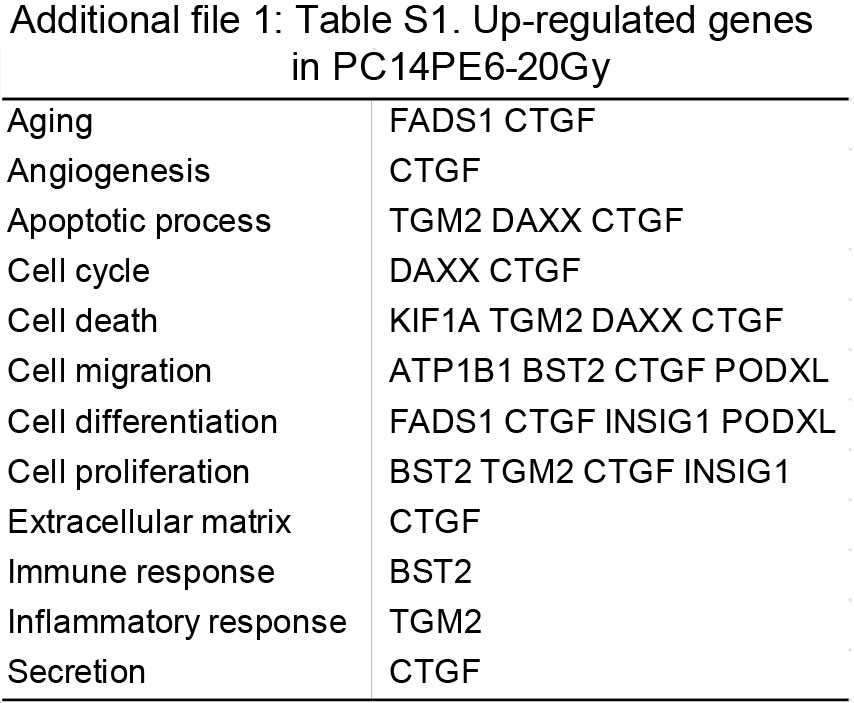


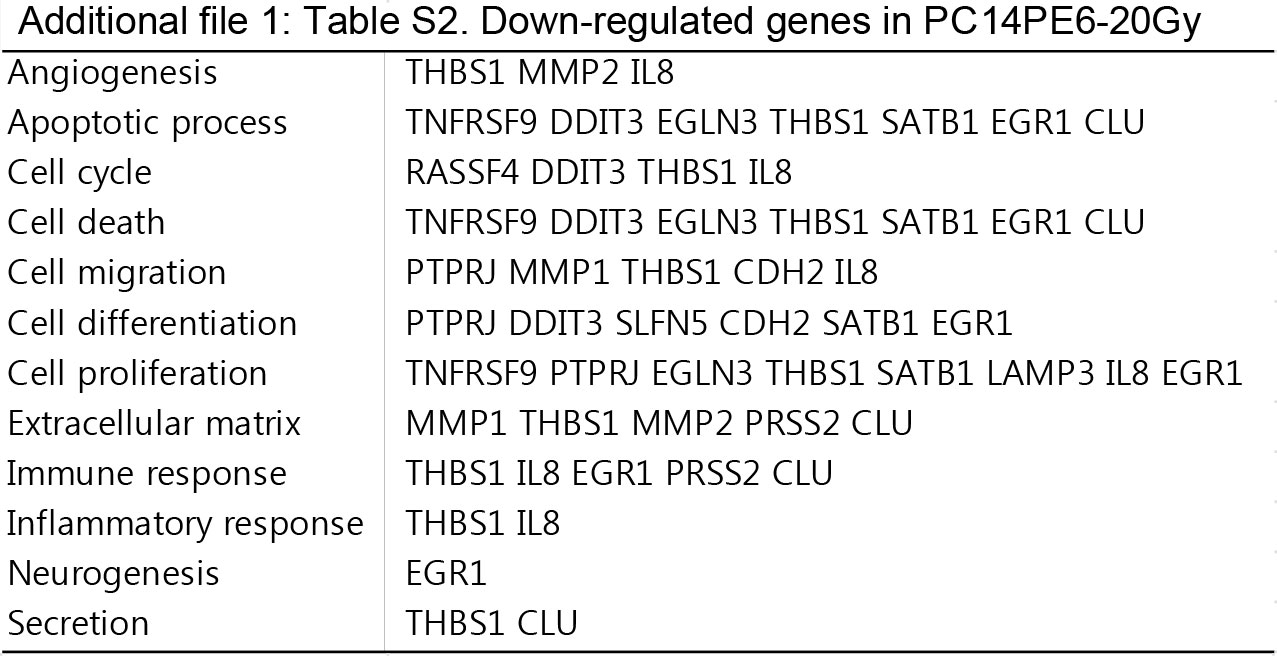


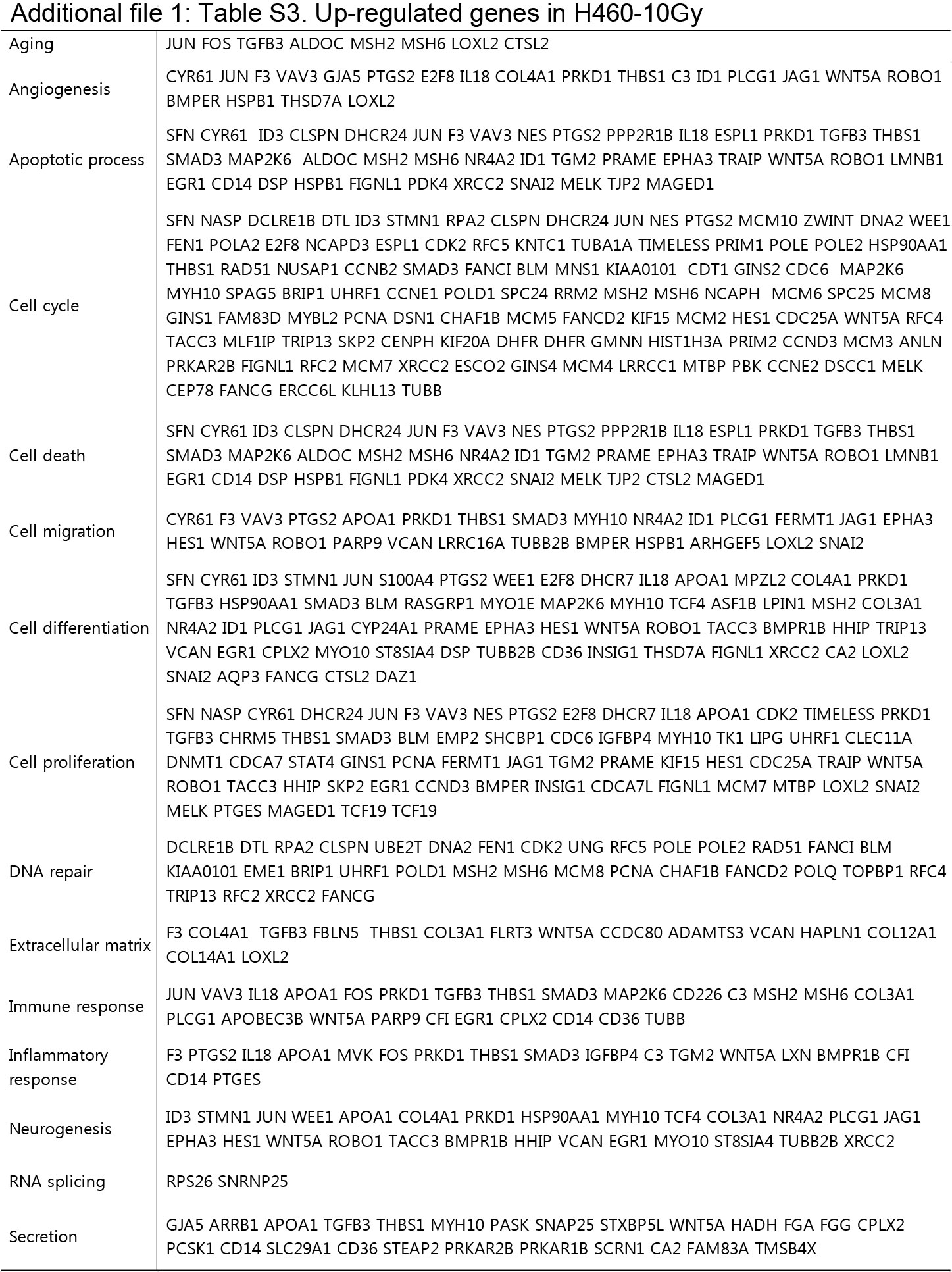


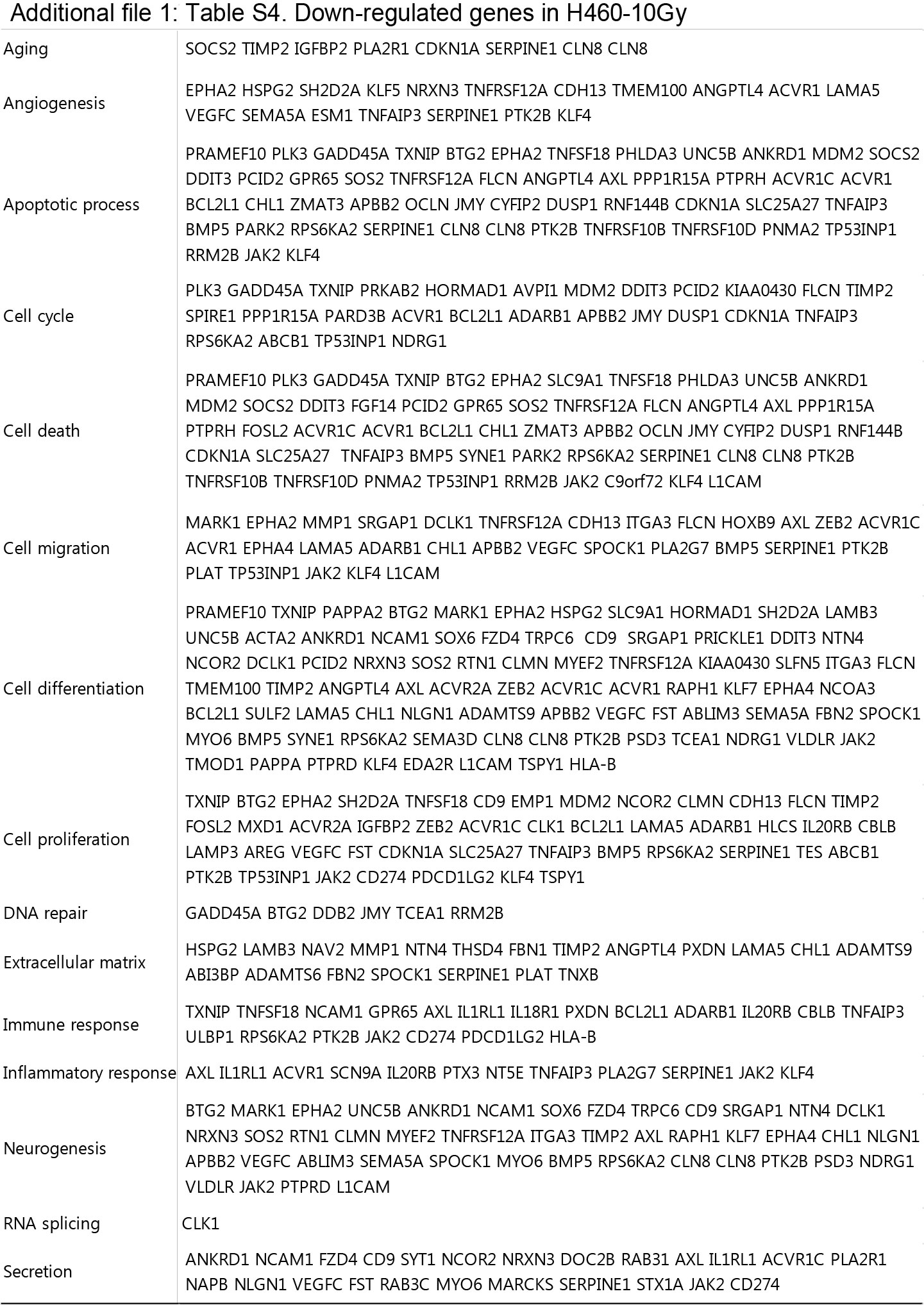


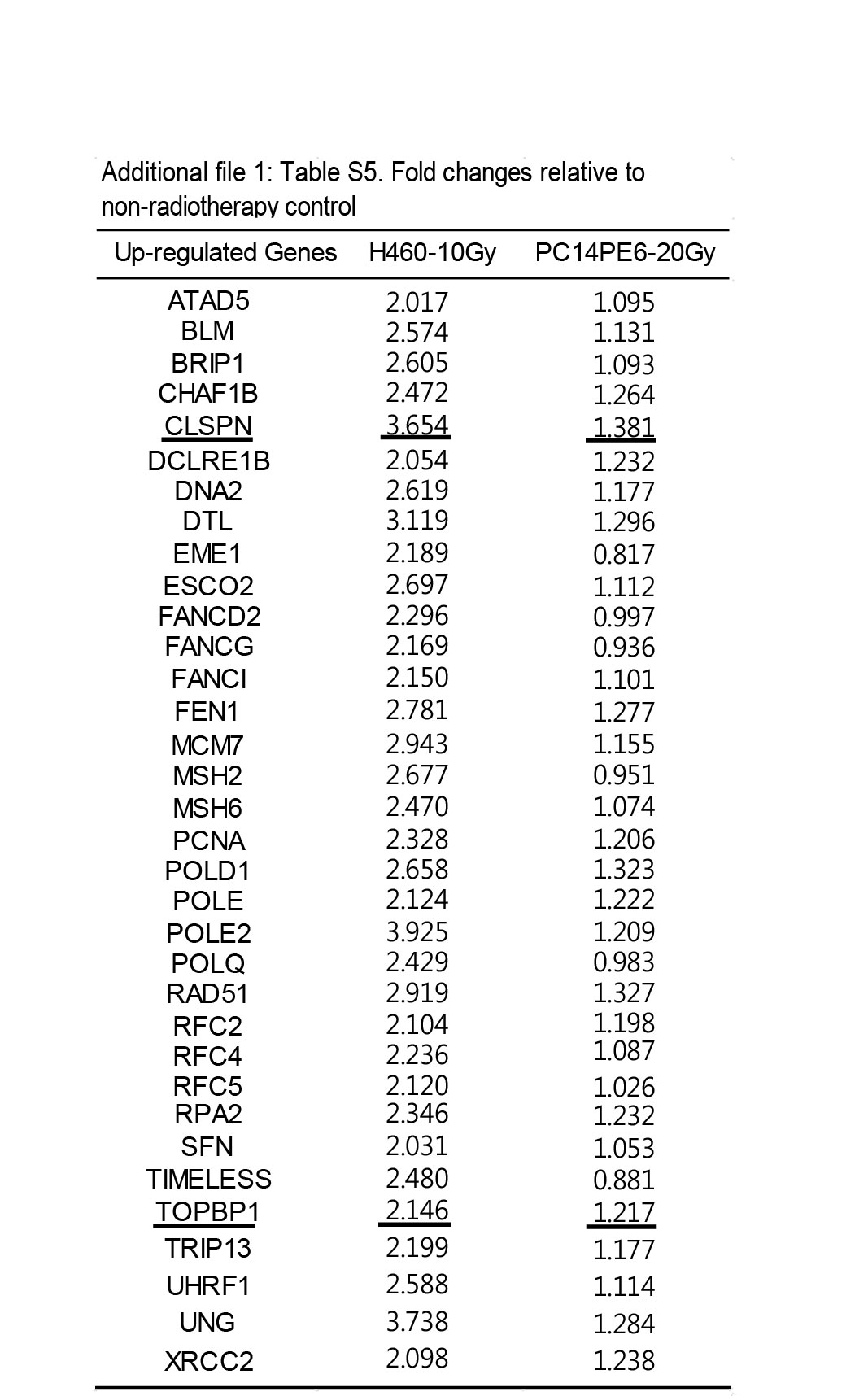


**Additional file 1: Figures**

**Additional file 1: Figure S1. Depletion of TopBP1 or Claspin in PC14PE6 cells.** To knockdown the target genes, the radio-resistant PC14PE6/shTopBP **(A)** and PC14PE6/shClaspin **(B)** cells were treated with doxycycline (1 mg/ml) for 72 hours. Total RNA was isolated from selected cell lines and mRNA levels were quantified by qRT-PCR. The mRNA levels of each gene were normalized with GAPDH mRNA level, and the fold change in target mRNA represents the relative value obtained from shRNA-treated and the control groups. *, *P*-value < 0.001. **(C)** Immunocytochemistry with TopBP1 and γ-H2AX antibodies. Cells were irradiated at 10 Gy. After 2 hours, cells were fixed and immunostained with TopBP1 and γ-H2AX antibodies. The γ-H2AX was used as a marker for damages from double stranded breaks of DNA.


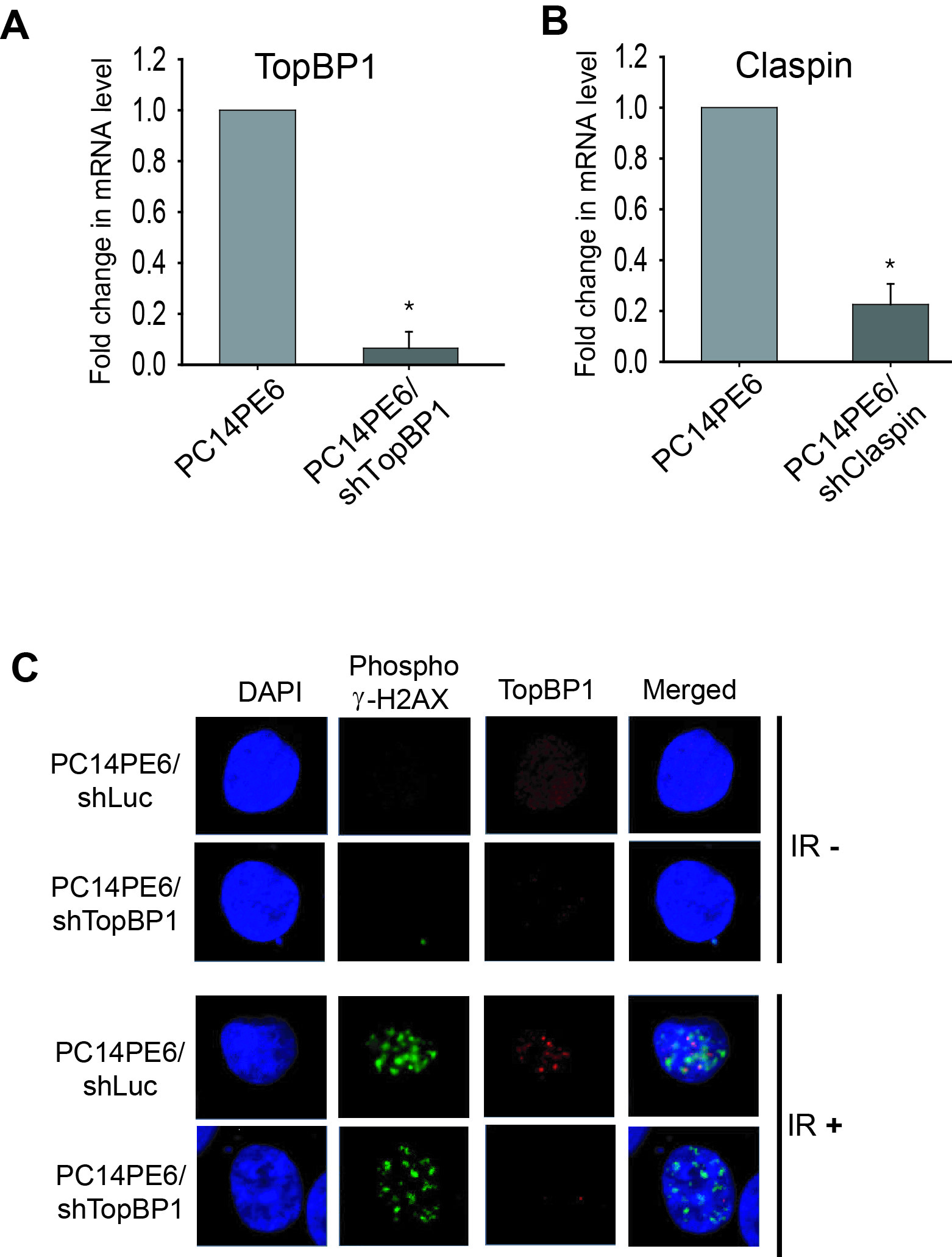


**Additional file 1: Figure S2. Radiosensitivity of lung cancer cell lines (H23, H460, and PC14PE6).** Clonogenic survival assays were performed with ionizing radiation (IR) treatment as indicated. U2OS, human osteosarcoma cells were used as the control. PC14PE6 cells are highly resistant and H23, H460, and U2OS cells are sensitive to IR. Values are from at least three independent experiments and error bars represent the standard deviation. *, *P*-value < 0.05; **, *P*-value < 0.001.


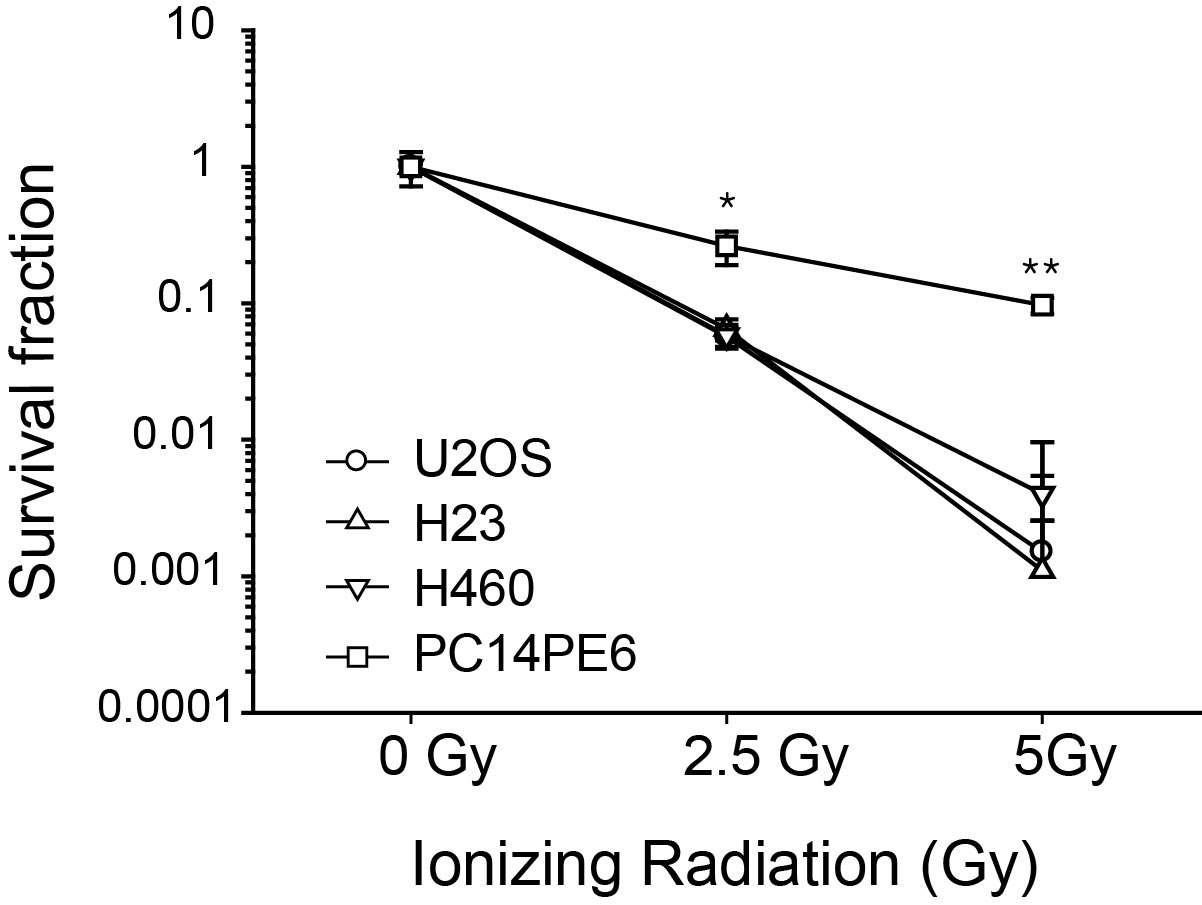

Supplement: Supplementary file 1 — Additional file 1: Supplementary Methods, Table S1-S5, and Figure S1-S2. (DOCX 2 MB) [file 12943_2014_1408_MOESM1_ESM.docx]
